# 

**Published:** 1884-10

**Authors:** 


### EDITORIAL.

### The Ohio State Dental Society.

The ninteenth annual meeting of this body will be held in the rooms of the Board of Trade, in Columbus, on Wednesday, October 29th, commencing at 10 o'clock A. M., and continuing three days, or until Friday noon. The following subjects for papers and discussion have been presented:

1—The Improvement in Artificial Dentures. 2—Affections and Treatment of the Active Organs of Mastication. 3—Therapuetics in Dental Practice. 4—Difficulties in Filling Teeth. 5—Pathological Condition of the Mucous Membranes of the Mouth—Cause and Treatment. 6—Food, Digestion, and Nutrition, in Relation to the Healthy Condition of the Teeth and Fluids of the Mouth. 7—Histology of Dental Tissues, as shown in the Process of Development.

An opportunity will be afforded for the exhibition of new instruments and appliances at such time as may be determined by the Society.

It is very desirable that there be a large attendance on the part of the membership, indeed of the whole profession of the State, as the interests of the Society require that a special effort should now be made for its support and progress. It is to be hoped that every member will come prepared to add to the interest of the occasion, and in this way benefit not only others, but himself as well. If every member would add something to the work, it would certainly very much increase the interest in, and efficiency of, the Society, as compared with former meetings. Let all appliances and instruments that are not in general use, and that are valuable, be brought, exhibited, and explained. Let each subject of this programme be thoroughly studied, so that each member will have something to say upon every subject presented.

An invitation to be present has been extended to quite a number of prominent members of the profession outside of the State, and it is confidently expected that they will be present and add much to the interest of the occasion. Some very important work devolves upon this meeting, especially with reference to amendments of the law regulating the practice of dentistry in this State. This was one of the first dental laws enacted, and it greatly needs revision and amendment, and it should without fail receive the attention of this meeting, and such arrangements be made as will secure the needed changes at the coming session of the Legislature. Let this matter also in the meantime be studied, so that all needed information may be at hand, that every one may see clearly what should be done, and be enabled to act properly and promptly in the matter.

The officers of the Society have put forth very considerable effort for making this meeting a success, and it is earnestly hoped that their endeavors willl be fully seconded by the membership. So let every one do what he may for a good and profitable meeting.

### Board of Examiners.

The annual meeting of the Ohio State Board of Examiners will be held in Columbus, Wednesday, October 29th, at 12o'clock M., for the examination of candidates, according to the requirements of the State law. All interested will please make a note of this, and be promptly on hand at the appointed time. It would be well for those who intend to apply for examination to send their names to the Secretary, Dr. F. H. Rehwinkle, Chillicothe, Ohio, before that time.

### CONTENTS.

### COMMUNICATIONS.

Reflex Pain........................................................................ 473

Chemistry............................................................................ 479

### PROCEEDINGS.

Proceedings and Discussions of the Twenty-fourth Annual Meeting of the American Dental Association.................................. 486

Salmagundi.......................................................................... 522

### EDITORIAL.

The Ohio State Dental Society.............................................. 525

Board of Examiners.............................................................. 526

Dentists' Benevolent Association........................................... 527

Obituary ............................................................................. 528

---

### The Dental Register,

A Monthly Magazine Devoted to the Interests of the

**DENTAL PROFESSION.**

*Terms Reduced to* $2.00 *Per Year. Single Copies,* 25 *Cents.*

**EDITED BY PROF. J. TAFT, D.D.S.**

—AND—

**Published by SPENCER & CROCKER, Ohio Dental and Surgical Depot.**

The volume commences in January and closes in December.

The Register being issued monthly is always fresh, therefore Indispensable to the practitioner who desires to keep posted up with the new inventions and improvements which are daily developed. Neither expense nor effort will be spared to give value and variety to its contents Articles will be illustrated with well executed engravings whenever they will add to the interest or assist to explanation.

Its extensive circulation and frequency of issue make it one of the BEST DENTAL ADVERTISING MEDIUMS in the United States.

All subscriptions must begin with January or July.

Local or special notices, five lines or less, will be inserted for $3 each insertion ; over five lines, 50 cts. per line.

All advertising bills payable in advance, or at furthest, after the issue of the first number containing the advertisement. All transient advertisements to be paid for in advance.

☞CONTRIBUTIONS SOLICITED.

Exclusively Editorial Communications should be addressed to the Editor.

The latest number of the Register may be ordered with or without other goods at any time, and all letters should be addressed to

**SPENCER & CROCKER, Ohio Dental & Surgical Depot, Cincinnati, O.**

